# Plant‐derived environmental DNA reveals fine‐scaled community differentiation in grassland arthropods

**DOI:** 10.1002/eap.70147

**Published:** 2025-12-19

**Authors:** Lisa Mahla, Juliana Becker, Lea Groß, Anna‐Sophie Tiltmann, Susan Kennedy, Henrik Krehenwinkel

**Affiliations:** ^1^ Department of Biogeography Trier University Trier Germany; ^2^ Faculty of Mathematics and Natural Science University of Bonn Bonn Germany

**Keywords:** biodiversity, eDNA, fine‐scale differentiation, flower, grassland, monitoring, plant–arthropod interaction

## Abstract

The loss of plant diversity in grasslands is implicated as one of the main causes of arthropod decline. The loss of a single plant species can have a cascading effect on specialized arthropod species. It is thus critical to expand our understanding of plant–arthropod interactions. Detecting plant–arthropod interactions, however, has been difficult, as it requires the observation of individual plant visits. A possible solution to this problem is offered by environmental DNA (eDNA) analysis. Here, we test the utility of eDNA to detect fine‐scaled community differentiation in grassland arthropods in Germany. Based on eDNA from 13 plant species, we explore community differentiation between plant species, and between flower and green parts of individual plants. We show that eDNA successfully recovers extremely fine‐scaled community differentiation. Plant species, as well as plant compartment, emerge as major drivers of arthropod community composition in grasslands, with the differentiation being particularly pronounced in herbivorous arthropods. Terrestrial eDNA on plants thus appears to be deposited in a very localized fashion, making this tool ideally suited to detect very fine‐scaled community differentiation. Considering the high specificity we observe in our analysis, our results highlight the necessity of integrating vegetation surveys into future monitoring of arthropod communities.

## INTRODUCTION

Plant–arthropod interactions are fundamental to the functioning of terrestrial ecosystems, supporting essential processes such as pollination, herbivory, seed dispersal, and nutrient cycling (Meyer et al., 2011; Ollerton et al., 2011; Seastedt & Crossley, 1984). The complex relationships between plants and their associated arthropod communities not only maintain biodiversity but also provide ecosystem services that are crucial to human well‐being (Brown et al., 2016; Ollerton et al., 2011). Global arthropod populations are experiencing significant declines (Hallmann et al., 2017; Seibold et al., 2019; van der Sluijs, 2020; Wagner et al., 2021), particularly in grassland habitats (Hallmann et al., 2017; Seibold et al., 2019), which are among the most biodiverse and vulnerable ecosystems on Earth (Bullock et al., 2011; Dengler et al., 2020). One assumed reason for the decline of grassland arthropods is a reduction in plant diversity (Borer et al., 2012; Haddad et al., 2009; Schaffers et al., 2008; Simons et al., 2014). Plant‐associated arthropod communities can be highly specialized, with many unique arthropod species occurring on each plant species (Novotny et al., 2006). A loss of an individual plant species at a site may thus be followed by extinctions of multiple arthropod species. And even different plant parts, such as flowers, stems, roots, or leaves, can host highly distinct arthropod communities (Wardhaugh et al., 2012; Weber et al., 2024). Hence, within a single plant, there can be significant microhabitat heterogeneity influencing arthropod diversity (Schoonhoven et al., 2005). This spatial variation in habitat use has important implications, particularly in managed ecosystems such as grasslands. Practices like early mowing, which alter plant structure, could disproportionately impact flower‐associated arthropod communities (Berger et al., 2024; Proske et al., 2022; Simons et al., 2014), potentially threatening specialized species and disrupting their ecological roles. Understanding how habitat characteristics, plant species diversity, and individual plant structures influence arthropod diversity is thus key to gaining insights into the health and functioning of grassland ecosystems. In turn, this information can enable predictions about and appropriate responses to insect decline. However, the detection of plant–arthropod associations is very laborious, as it requires the direct observation of interactions (Opp & Prokopy, 2014). Hence, it is rarely included in conventional arthropod monitoring schemes.

Recently, environmental DNA (eDNA) metabarcoding has emerged as a rapid and simple approach for the detection of arthropod–plant interactions without the need for direct observation or collection (Ji et al., 2013; Krehenwinkel et al., 2024; Stothut, Kühne, et al., 2024; Stothut, Mahla, et al., 2024; Taberlet et al., 2018; Thomsen & Sigsgaard, 2019; Weber et al., 2024). Arthropods leave DNA traces when interacting with a plant (Wagner et al., 2021). These traces can be enriched by metabarcoding to recover plant‐ or plant compartment‐specific arthropod communities (Krehenwinkel, Weber, Broekmann, et al., 2022; Macher et al., 2023; Thomsen & Sigsgaard, 2019; Weber et al., 2024). While eDNA in aquatic ecosystems freely disperses through the water and reflects a broad range of species (Sales et al., 2020; Yang et al., 2021), plant‐derived eDNA is believed to primarily originate from organisms directly interacting with the plant. Hence, its deposition is expected to be very localized (Krehenwinkel, Weber, Broekmann, et al., 2022; Stothut, Kühne, et al., 2024; Stothut, Mahla, et al., 2024), making terrestrial eDNA an excellent tool to explore extremely fine‐scaled community differentiation between plant species and even compartments within individual plant specimens.

However, it remains to be tested whether arthropod eDNA can be deposited on plant material by means other than direct interaction. If so, eDNA could lead to false‐positive detections of interactions. One factor that could influence the localized distribution of eDNA on plant surfaces is aerial eDNA. Previous studies have demonstrated that it is possible to capture eDNA from airborne particles, including those from insects, by filtering air (Métris & Métris, 2023; Roger et al., 2022). This airborne eDNA could drift and become mixed with plant‐associated eDNA, leading to the spurious detection of arthropods on various plants and plant parts, and blurring true biotic interactions. Considering this background, we here test the utility of plant‐derived eDNA to detect fine‐scaled community differentiation in grassland arthropods.

We performed two experiments in grasslands in the German city of Trier. First, we sampled eDNA from 13 common grassland plant species across six different sites. Second, we conducted a more detailed investigation of eDNA present on specific plant parts, comparing the green tissues (stems and leaves) and inflorescences for six selected plant species across two sites. Using the resulting datasets, we tested the hypotheses that (1) plant species diversity is a main driver of community differentiation in grassland arthropods. Consequently, we expect to detect numerous plant‐species‐specific arthropod taxa. We also hypothesize that (2) even at the level of individual plant compartments, significant community differentiation will be detected. Different arthropod species will be detected on leaves versus flowers of individual plants. Many plant species possess complex chemical defenses against herbivorous arthropods. The arthropod herbivores in turn must adapt to overcome the plant's defenses. This can lead to pronounced specialization of herbivorous arthropods to their host plants. We thus also hypothesize that (3) arthropod community differences between individual plant species or plant compartments will be more pronounced in herbivorous taxa than in non‐herbivores. As an alternative hypothesis, we postulate that (4) aerial eDNA may lead to a lack of differentiation of arthropod communities detected on plants. If aerial eDNA is deposited on a limited spatial scale, we would expect to observe only weak differentiation between plant species and no differentiation between compartments on an individual plant.

## METHODS

### Study area and sampling design

**FIGURE 1:**
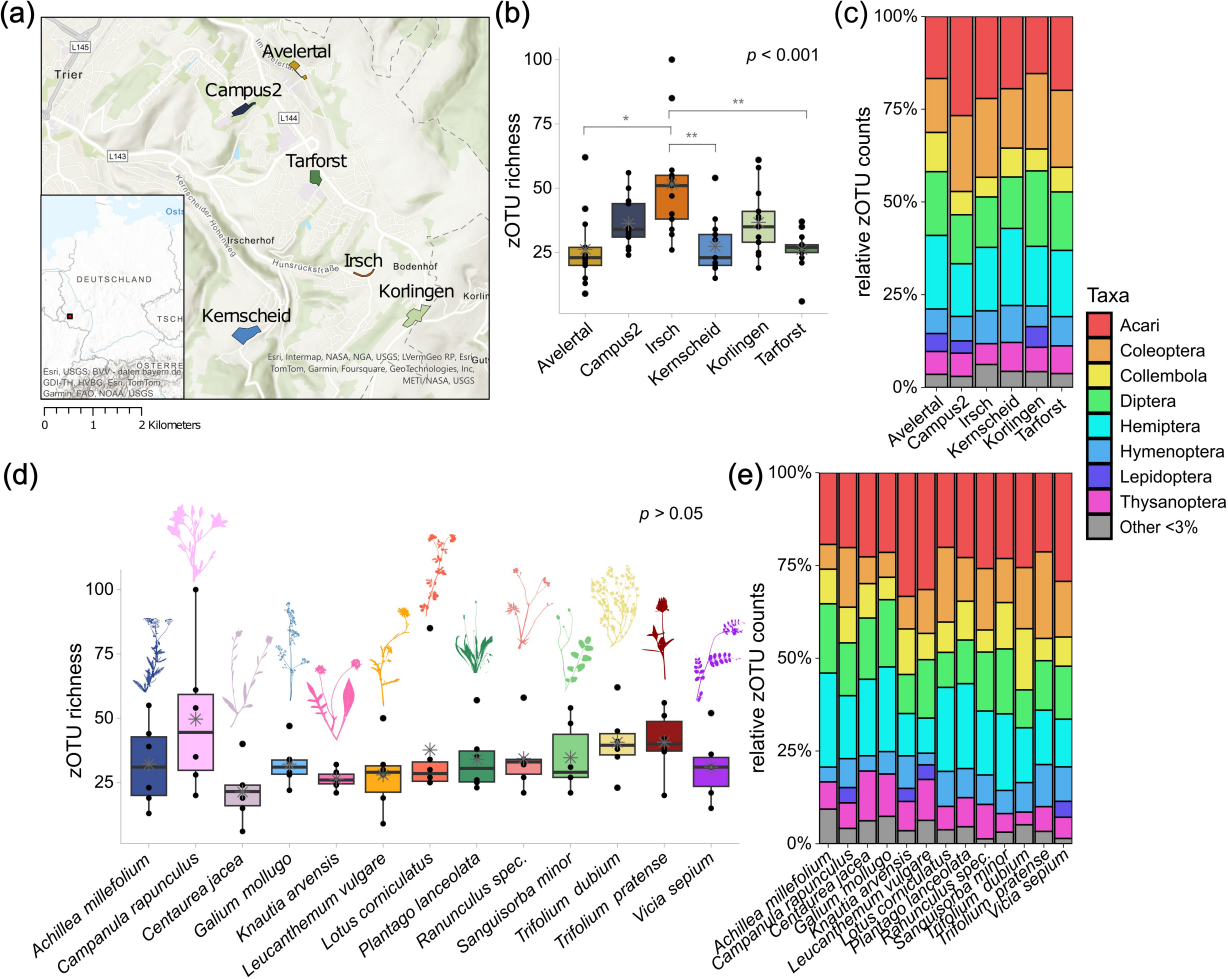
This overview graphic of the dataset of the whole‐plant specimens shows (a) the different sites analyzed in the study. The zero‐radius operational taxonomic unit (zOTU) richness (b) of each site and (d) of the 13 complete collected plant species is shown as boxplots with error bars for the 95% CI; the mean value is shown with an asterisk; significant differences are calculated with Kruskal‐Wallis rank sum test (*p*) and for the sites the pairwise‐Wilcoxon's test (holm correction) (**p < 0.01, *p < 0.05) for the significant differences. The taxonomic composition of (c) each site, as well as of (e) each plant species is shown in a bar chart of percentage of the different zOTU counts per order, with colors representing the different taxa/orders. All Acari and Collembola orders were grouped together and orders making up less than 3% of the number of zOTUs were grouped together in “Other <3%” (gray). Plant silhouettes used for visualization purpose were created by the author Lisa Mahla.

The study was conducted across six grassland sites in and around Trier, Rhineland‐Palatinate, Germany (Figure 1a). When selecting the sites, we did not explicitly include the intensity of land use or the surrounding habitat types as factors; nevertheless, differences in land‐use type and neighborhood structure, the degree of agricultural and urban influence in the immediate and surrounding areas as well as altitude and openness were noted (Appendix S1: Table S1). Despite some differences between the sites, the aim was to examine ecologically similar sites. Therefore, we chose sites with similar vegetation. This enabled us to select the same target plants for all the sites, thereby ensuring diversity in flowering periods, colors, families, growth heights, and life cycles (Appendix S1: Table S2). These different attributes were selected to include a range of plant characteristics in the analysis, to identify which ones may be particularly important. Flower color was chosen as it is well known to influence attraction and visual perception in many insect taxa. The lifeform classification reflects growth and overwintering strategies; both can affect the temporal availability of resources and habitat structures. The same is true of growth height. Sampling occurred from June 5 to 7, 2023, following a minimum of three rain‐free days to prevent eDNA wash‐off (Valentin et al., 2021).

### Plant sampling procedure and processing

Target plant individuals were sampled across each grassland site depending on their natural occurrence. Although the intention was to collect samples from different parts of each site to avoid spatial clustering, the final selection was limited by the local abundance and distribution of each species. Some species, such as *Knautia arvensis* (L) Coult., s. str, were widespread and could be sampled throughout the site. Others, such as *Trifolium dubium* Sibth., occurred only in small patches, sometimes near the edges of the site. At the Irsch site, sampling was limited to a remaining flower strip due to recent mowing. At each site, a total of 25 g per plant species of fresh mass from above‐ground components of 13 plant species were collected (whole‐plant specimens). The plant material was carefully picked just above the soil surface. Fresh gloves were used for each species, and care was taken not to touch the surrounding vegetation. Additionally, a more detailed sampling was conducted at two sites (Avelertal and Tarforst). For this, four replicates of six plant species (*Achillea millefolium* L., *Centaurea jacea* L., *Knautia arvensis*, *Leucanthemum vulgare* agg., *Sanguisorba minor* Scop., and *Trifolium pratense* L.) were collected The flower heads (referred to as “flowers”) were separated from the green parts containing the stems and leaves (referred to as “stems”) of each individual plant, with new gloves being used to minimize cross‐contamination between plant compartments. To get a minimum of 5 g flowers, 10 individuals were collected per replicate, except for *K. arvensis* where five individuals were sufficient. The two parts of the plant were placed in separate ziplock bags. These bags were then weighed and 10 times their mass in deionized water was added and shaken thoroughly to wash off as much eDNA as possible. We routinely use this water and have tested its suitability (Stothut, Kühne, et al., 2024; Stothut, Mahla, et al., 2024; Weber et al., 2024). The water (without the plant tissue) was then transferred into new ziplock bags and stored at −23°C until further processing. To rule out potential contamination in the field, negative water control samples were collected. For each control sample, 250 mL of deionized water (drawn from the same canister as the water used for rinsing the plant samples) was poured into a new Ziploc bag identical to those used for the plant samples. The bag was shaken, after which the liquid was transferred to a second clean bag and stored at −23°C. This procedure was carried out at two locations in the field, corresponding to the two separate water fillings from the canister. This was done in order to test each batch of water for potential contamination. These field controls were then processed in the same manner as the samples. Subsequently, samples and controls were filtered in the laboratory using Cellulose Nitrate (CN) Membrane Filter Cups with a pore size of 0.45 μm (Sartorius Stedim Biotech GmbH, Goettingen, Germany) as performed by Weber et al. (2024), Stothut, Kühne, et al. (2024), and Stothut, Mahla, et al. (2024). Clogged filter membranes were replaced by a second CN membrane after a maximum filter time of 3 h. The filter membranes for each sample were then combined in the isolation process to form one DNA extract per sample.

### eDNA extraction and amplification

DNA extraction was performed using the DNeasy Blood and Tissue Kit (Qiagen, Hilden, Germany) with slight modifications. Membranes containing the samples were placed in 2‐mL tubes along with 0.035–0.04 g of silica zirconium beads. Following the addition of 540‐μL ATL Buffer, the samples were beadbeaten at 1500 rpm using the Spex 1600 miniG (SPEX, Metuchen, New Jersey, USA). After lysis and centrifugation, 600 μL of ethanol was added to each filter. Subsequently, a volume of 50‐μL DNA extract per sample was obtained and stored at −23°C. An isolation control containing only the reagents and beads was processed in the same way as the samples and field controls.

Using a polymerase chain reaction (PCR) we amplified the extracted DNA of 174 samples, two field controls, two isolation controls, and two PCR controls with the Qiagen Multiplex PCR kit and the primer pair fNoPlantF_270 (forward primer: RGCHTTYCCHCGWATAAAYAAYATAAG) and mlCOIintR_W (reverse primer: GRGGRTAWACWGTTCAWCCWGTNCC), targeting a fragment length of 116 bp of the COI gene (Krehenwinkel, Weber, Künzel, & Kennedy, 2022). PCR was conducted with one replicate in a 10‐μL reaction containing 1‐μL template DNA and a total of 1‐μL 10‐μM primers at a denaturing temperature of 94°C, an annealing temperature of 46°C and an elongation temperature of 72°C for 36 PCR cycles, following the manufacturer's protocol. Subsequently, Illumina TruSeq libraries were generated (Lange et al., 2014), and the indexed samples were pooled in approximately equal amounts based on gel band intensity.

### Sequencing and data preparation

The pool was purified with 1× magnetic beads (AMPure XP, Beckman Coulter, California, USA) and sequenced on an Illumina MiSeq (Illumina Inc., San Diego, California, USA) with 20,000 reads per sample using a 600‐cycle V3 kit. The field control as well as negative controls of the DNA isolation and PCR were sequenced as well. After sequencing, the raw sequence reads were demultiplexed using the MiSeq Local Run Manager (Illumina Inc.) with no mismatches allowed. Forward and reverse reads were then merged using PEAR (Zhang et al., 2014) with a minimum overlap of 50 bp and a minimum quality score of Q20. The merged reads were quality filtered (≥90% bases with ≥Q30) and converted to fasta files using the FASTX‐Toolkit (Gordon & Hannon, 2010). The processed reads were then trimmed of primer sequences, leaving a fragment of approximately 64 bp, and dereplicated using USEARCH (Edgar, 2010). They were then clustered (denoised) into zero‐radius operational taxonomic units (zOTUs), including a chimera‐removal step, with a minimum size of eight occurrences. The zOTUs were then searched against the NCBI database using BLASTn, retaining the top 10 results (Altschul et al., 1990). A Python script was used to assign the taxonomy to the Blast output (Schöneberg, 2025). Non‐arthropod hits (e.g., Fungi and Rotifera) were removed, and the first hit for each zOTU was used for creating the table.

Further analysis involved filtering taxonomically annotated zOTUs to retain only those meeting specific criteria. We kept only taxonomically annotated zOTUs with a length ≥53 bp and 85% reference hit to the database and excluded zOTUs from the order Decapoda (four zOTUs), Cumacea (one zOTU), Pantopoda (one zOTU), Cyclopoida (one zOTU), and Amphipoda (six zOTUs). Finally, the data were filtered for potential cross‐contamination (e.g., by index bleeding) by identifying the highest read count found in the control (10 reads), and then setting all occurrences of ≤10 reads to 0. Samples that did not yield more than one zOTU and one sample detecting only Acari were removed, resulting in 78 samples for the whole‐plant specimens and 89 samples for the plant compartments (46 Stem samples, 43 Flower samples). When looking directly at different taxa, a filtering threshold was used at a percentage match to the reference database of 95% for family level, 98% for genus level, and 99% for species level. For analyzing arthropods' feeding guild and life history, only those zOTUs with ≥98% match to the database (treated as accurate genus‐level identifications) were included. Herbivorous arthropods were categorized based on strict annotations as gall builder, miner, chewer, or sap sucker. Other arthropods, including parasites, nectarivores and palynivores, were grouped as “non‐herbivores.” The feeding type and herbivore specialization were assigned using the database of Ellis (2001–2025) as well as primary literature. The preferences were primarily based on larval life history. Details are available in our related data deposit (Mahla et al., 2025 at https://doi.org/10.6084/m9.figshare.28062344.v1, file name: Ecological_annotation.xlsx).

### Statistical analysis

Statistical analysis was conducted using R version 4.2.2 and RStudio version 2022.7.2.576. The package dplyr (Wickham et al., 2019) was employed for data management and plotting and the package vegan (Oksanen, 2010) for statistical tests. Alpha diversity was assessed using zOTU richness, while beta diversity was analyzed using binary Jaccard dissimilarity with vegdist. Mean zOTU richness differences were evaluated using a Kruskal–Wallis test and pairwise Wilcoxon's test (holm correction). Clustering of arthropod communities and effects of different plant attributes (flower color, plant height, lifeform) were tested using permutational multivariate ANOVA (PERMANOVA), pairwise PERMANOVA, and analysis of similarities (ANOSIM) using the adonis2() function and pairwise.adonis() function with permutations = 999, method = “jaccard,” and binary = True. The results were visualized with nonmetric multidimensional scaling using the metaMDS() function (NMDS, *k* = 2, jaccard, try max = 1000). Bipartite networks were created with taxonomically annotated and filtered (99% for species, 98% for genus, 95% for family) zOTUs for the complete collected samples using the function plotweb() with method = “cca” of the package bipartite(). The UpSet plot was created with the package UpSetR (Conway et al., 2017). For testing differences in herbivore and non‐herbivore arthropod communities, beta diversity (pairwise dissimilarities) was calculated as before. Species accumulation curves were created with the package iNext() (Appendix S1: Figure S5) and rarefaction curves were created for each sample (Appendix S1: Figure S6).

## RESULTS

A total of 9,036,230 raw reads were generated through sequencing. After quality filtering, 6,478,309 reads were mapped into 8281 zOTUs. Out of those, 2112 zOTUs were assigned to Arthropoda, representing a diverse array of 18 orders, 197 families, and 473 species. Additionally, 934 (436 herbivore, 498 non‐herbivore) zOTUs were ecologically annotated (98% hit to database), shedding light on the functional roles within these communities. Our filter steps left no more reads in the controls. In addition, 168 samples of the 174 initial samples remained for analysis, which on average had 26.9 (±1.22) arthropod zOTUs and 12,033 (±728) reads (Appendix S1: Figure S6).

### Arthropod communities at different scales

**FIGURE 2:**
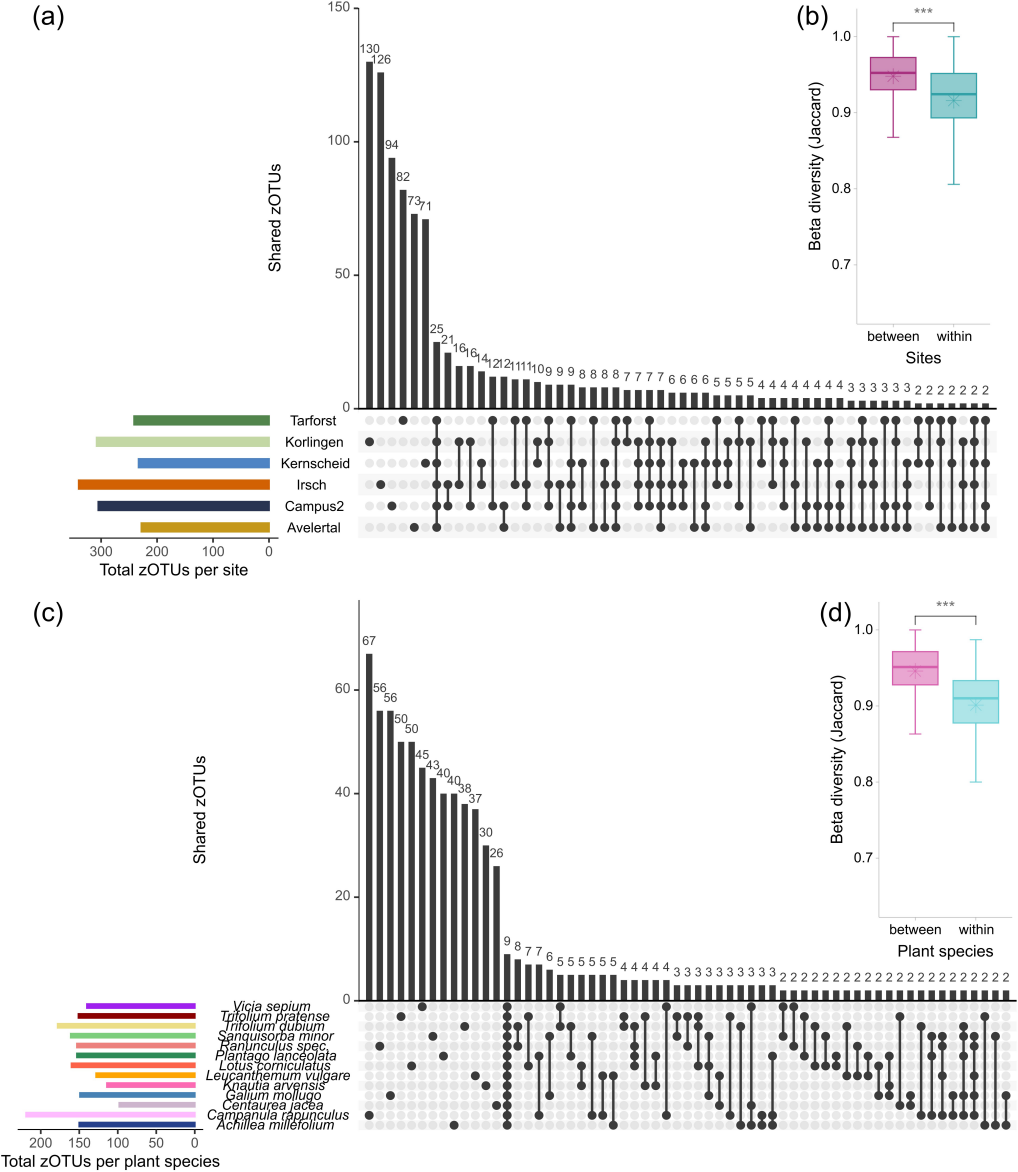
UpSet plot illustrating the distribution and overlap of zero‐radius operational taxonomic units (zOTUs) of arthropods at the analyzed sites (a) and plant species (c). The horizontal bars (colored by site or plant species) show the total number of zOTUs at each site or for each plant species, while the vertical bars show the size of the intersections (i.e., number of co‐occurring zOTUs) between the respective sites or plant species. Dots and lines below the bars indicate the sites or plant species involved in each intersection. Intersections with less than 2 zOTUs are not shown. The boxplot of Jaccard dissimilarity between different sites or within the same site (b) or plant species (d) shows the mean value (asterisk) and the significance values for the Wilcoxon's test (***p < 0.001).

Our investigation of the arthropod communities in grasslands revealed interesting patterns of specialization at different levels. At the broadest level of analysis, differences in arthropod zOTU richness were evident between study sites (Kruskal–Wallis *p* < 0.001), with Irsch and Korlingen having the highest richness (Figure 1a). Further differences could be shown by the unique taxa/zOTUs at each site (Figure 2a, Appendix S1: Figure S1), which varied between 29% and 37% for each study site. Beta diversity also showed that the Jaccard dissimilarity between plant species at each site was significantly lower than the Jaccard dissimilarity between plant species from different sites (Figure 2b). In particular, the Irsch site was found to have the lowest mean Jaccard dissimilarity within the site (0.84). However, the arthropod communities showed overall large overlaps between sites (Figure 2a, Appendix S1: Figure S1), and very similar order composition, with the most zOTUs assigned to orders/taxonomic groups Acari, Hemiptera, Coleoptera, and Diptera (Figure 1c). When examining the effects of environmental conditions (e.g., openness, agricultural influence) and other site‐specific factors (Appendix S1: Table S1), no clear trends could be identified regarding the arthropod communities.

Zooming in on individual plant species, we found no significant differences in alpha diversity (Kruskal–Wallis *p* > 0.05, Figure 1d) among the 13 different species. The order composition was also very similar in all plant species (Figure 1e). However, beta diversity analysis unveiled significant differences in community composition between plant species (ANOSIM: *R* = 0.59, *p* < 0.001; PERMANOVA: *R* ^2^ = 0.23, *p* < 0.001). The mean Jaccard dissimilarity between each of the plant species was significantly higher than within the same species (Figure 2d, Wilcox test *p* < 0.001). Notably, various arthropod plant specialists were identified, demonstrating host‐specific associations with plants such as *Ozirhincus millefolii* on *A. millefolium* and *Protapion trifolii* on the genus *Trifolium* (Appendix S1: Figure S2). This specialization is also evident in the number of unique zOTUs per plant species (Figure 2c, Appendix S1: Figure S1). For almost all plant species, 25% of the zOTUs detected at that plant were unique. Furthermore, 50% of the zOTUs per plant species were also found on only two additional species. Most of those unique zOTUs occurred only in one sample, while a few were detected in every sample of the plant species (Appendix S1: Figure S1).

**FIGURE 3:**
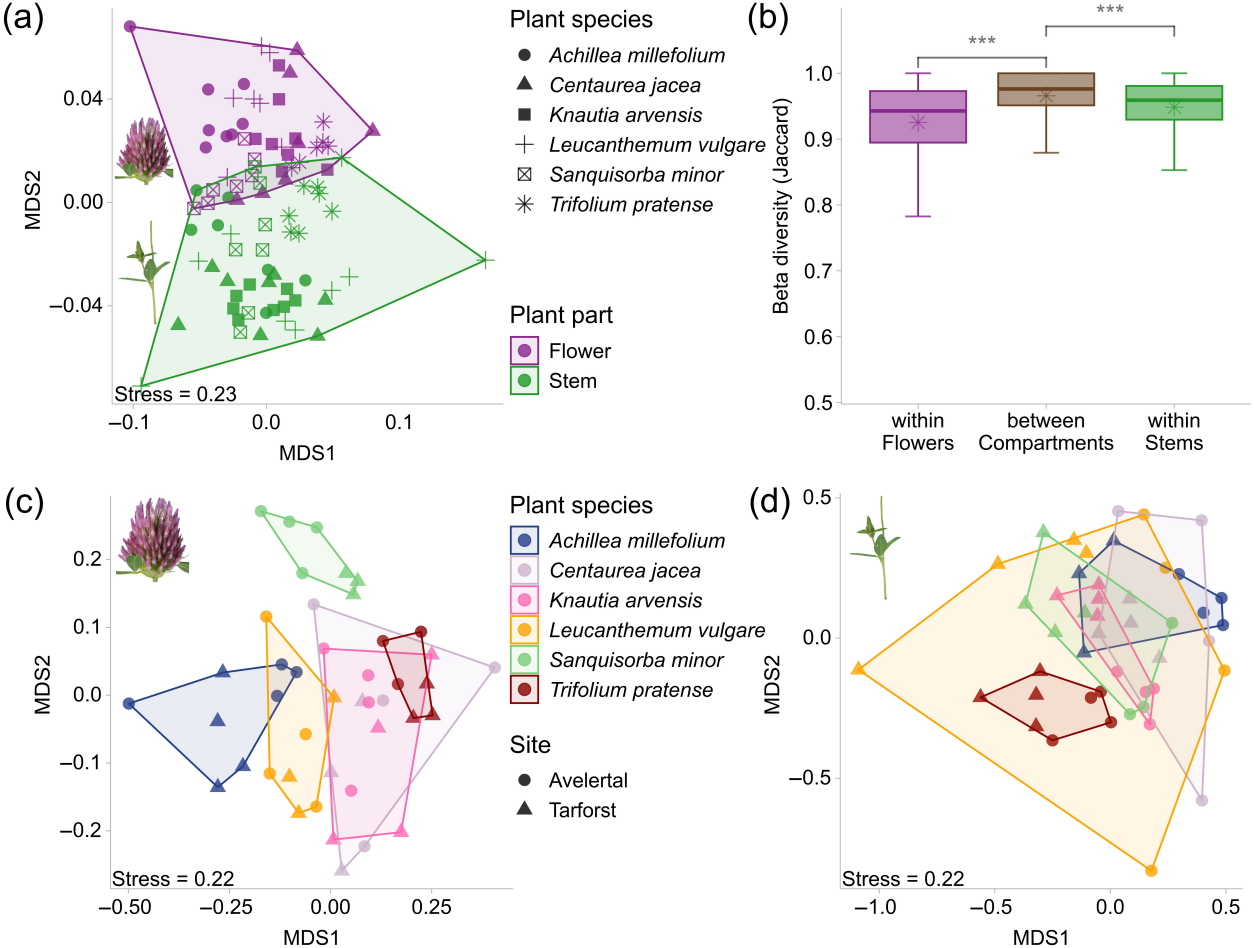
The different arthropod communities in the two compartments studied are presented with (a) an nonmetric multidimensional scaling (NMDS) plot (shapes representing the different plant species) and (b) a boxplot of Jaccard dissimilarity, showing mean value (asterisk) and significance level calculated with Wilcoxon's test (***p < 0.001). The association of the individual plant species in the compartments is shown as NMDS for (c) flowers and (d) stem samples. *Trifolium pratense* photo credit: Jonas Henn.

At the finest scale, examination of different plant compartments revealed further differentiation. While zOTU richness did not significantly differ between plant parts (Appendix S1: Figure S3, pairwise‐Wilcoxon's test *p* = 0.2549), arthropod communities exhibited significant separation between the green parts (stem) and the inflorescences (flowers) (Figure 3a, ANOSIM: *R* = 0.29, *p* < 0.001). Notable differences in the distribution of taxa were observed (Fischer test: *p* < 0.01), with Acari accounting for a significantly higher proportion in stems than in flowers (Appendix S1: Figure S3, Acari: *p*.adj <0.01). When analyzing the individual plant parts independently, further differences between these compartments became apparent. Flowers showed a clearer clustering of the arthropod community by plant species (Figure 3c, ANOSIM: *R* = 0.6, *p* < 0.001) than the stem samples (Figure 3d, ANOSIM: *R* = 0.33, *p* < 0.001). The Jaccard dissimilarity was lower within the same compartment than between different compartments (Figure 3b), indicating a distinctive arthropod community for each compartment.

### Testing for different influences on arthropod assemblage

**FIGURE 4:**
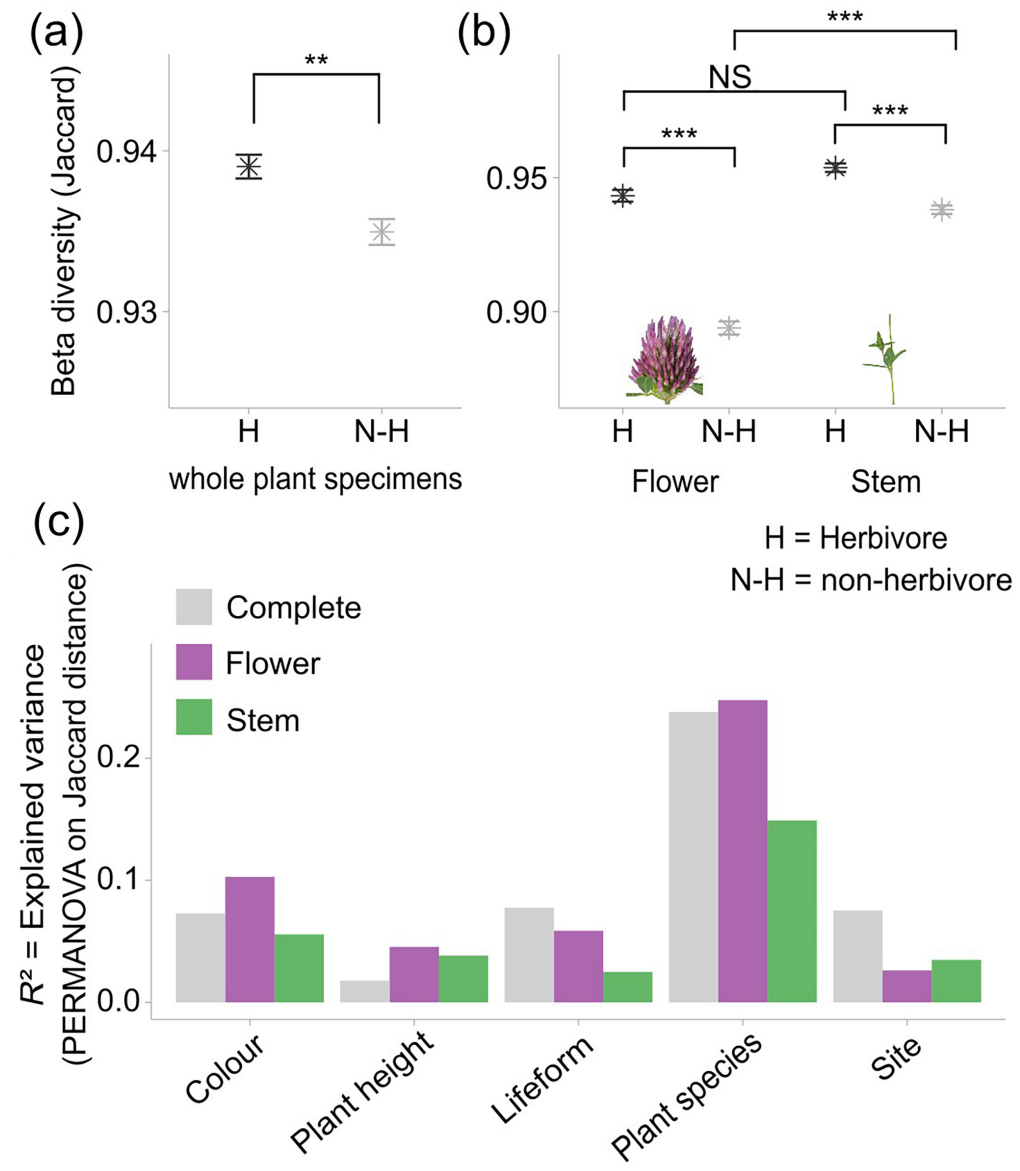
Pairwise Jaccard dissimilarity between communities of herbivorous (H) versus non‐herbivorous (N‐H) arthropods for (a) whole plants and (b) flower and stem samples separately. The significance levels from Wilcoxon's test are represented in the diagrams (NS = not significant, **p < 0.01, ***p < 0.001). Herbivores include mining, galling, plant chewing, and sucking arthropods while non‐herbivores include pollinators, predators, and other feeding guilds. The influence of the different plant characteristics, plant species, and sites on community composition was measured using permutational multivariate ANOVA (PERMANOVA) (Adonis2). The bars (c) indicate the calculated *R* ^2^ values for each term in the model, based on Jaccard distances and *p* value adjustment using the Holm method. Results are shown separately for the whole‐plant specimens as well as for the flower and stem samples. Only significant terms (*p* < 0.05) are displayed. Detailed descriptions of the analyzed plant attributes are provided in Appendix S1: Table S2. *Trifolium pratense* photo credit: Jonas Henn.

The role of different plant attributes for the plant‐associated arthropod community was found to have no influence above 10% (Figure 4c, Adonis: *R* ^2^ < 0.1). Plant characteristics, including growth height and life form, were found to have no effect on the arthropod communities. The site identity was identified as an explanatory factor, with a relatively low *R* ^2^ value (Adonis: *R* ^2^ ~ 0.08). The only variable to demonstrate an *R* ^2^ value exceeding 0.1 for the flower samples was flower color (Figure 4c). A slight pattern by color can also be observed in the NMDS of the flower samples (Figure 3c). The two species with white flowers (*A. millefolium*, *L. vulgare*) are grouped next to each other and the species with pinkish red flowers (*T. pratense*, *C. jacea*, and *K. arvensis*) are also clustered together, sometimes overlapping each other. The rather inconspicuous (green–brown) flowering meadow button (*S. minor*), which is also the only wind‐pollinated species, showed no overlap with the other species in the NMDS. The only variable that appears to have an explanatory role is the plant species itself. An *R* ^2^ of 0.15 was determined for the plant species of the stem samples and an *R* ^2^ of over 0.20 for the total plant samples and flower samples, respectively.

### Comparing herbivore and non‐herbivore communities

The analyses of herbivores and non‐herbivores were conducted with approximately equal group sizes (436 herbivores, 498 non‐herbivores). Only the flower samples tended to show proportionally slightly more herbivorous arthropods than non‐herbivorous arthropods, and vice versa for the stems (Appendix S1: Figure S4). There was also no great influence of plant characteristics on herbivore or non‐herbivore communities within each compartment and on the whole‐plant specimens (Appendix S1: Table S3). However, there was a difference in the influence of the plant species. In the whole‐plant specimens, the influence was greater for herbivores (*R* ^2^ = 0.25) than for non‐herbivores (*R* ^2^ = 0. 20). The same was true for the flower samples (herbivores *R* ^2^ = 0.26, non‐herbivores *R* ^2^ = 0.18). The stem samples, on the other hand, showed only a slight shift in favor of herbivores (herbivores *R* ^2^ = 0.17, non‐herbivores *R* ^2^ = 0.15) (Appendix S1: Table S3).

This different pattern of arthropod community structure by plant species could also be revealed by the beta diversity between these two arthropod groups, as the Jaccard dissimilarity for the whole plants was significantly higher in herbivores than in non‐herbivores (Figure 4a). The same patterns were found for the individual compartments. The mean Jaccard dissimilarity between different plant species in flowers was significantly higher in herbivores than in non‐herbivores. The same was true for the stem samples (Figure 4b). The arthropod community composition for herbivores showed strong clustering by plant species, especially in the flower samples (ANOSIM, *R* = 0.6, *p* < 0.001) but also in the stems (ANOSIM, *R* = 0.4, *p* < 0.001). Pairwise analyses supported these results, with more significant trends calculated for flower samples and less frequently for stem samples. In the non‐herbivorous arthropods, some degree of community differentiation between plant species was also observed, but significantly less than in the herbivores (Appendix S1: Table S4).

## DISCUSSION

We used eDNA metabarcoding to investigate arthropod communities associated with grassland plants, focusing on how plant species and different plant compartments influence arthropod diversity and composition. Our results show that interactions between plants and arthropods can be effectively captured using eDNA, providing valuable insights into the fine‐scale distribution and specificity of arthropod communities in grassland ecosystems, and demonstrating the powerful potential of eDNA metabarcoding for ecological studies.

Plant species are an important driver of arthropod communities. This was demonstrated by a high number of unique arthropod detections for each plant species, including various known specialists. The distinct arthropod signatures associated with each plant highlight the strong dependency of certain arthropods on specific plants. However, only a few zOTUs were detected across all plant individuals and sites. While undersampling can affect data completeness, particularly in complex arthropod communities, we aimed to sequence deep enough to substantially reduce this issue. Consequently, some zOTUs may appear to occur exclusively on a single plant species, which is more likely to reflect ecological patterns, although a minor influence of residual sampling limitations cannot be entirely excluded. Aside from plant species identity, other plant attributes were analyzed to test for an influence on arthropod community composition. Diverse vertical structures and life types can lead to an increase in arthropod diversity (Dennis et al., 1998; Lengyel et al., 2016). Contrary to expectations, no considerable influence of structural variables or plant species traits was observed.

However, the individual sites differed significantly from one another in terms of both their alpha and beta diversity, indicating a strong turnover in arthropod communities across space. Despite having broadly similar vegetation compositions, the sites varied in size and in the characteristics of their immediate and wider surroundings, as well as in terms of the presence of hedgerow structures. Such differences can create distinct environmental conditions and microclimates, influencing species composition (Chikowore et al., 2021; Joern & Laws, 2013). Despite this, the sites were ecologically very similar overall, and these differences were relatively minor compared to the shared general habitat characteristics.

At the site Irsch, however, the available habitat was reduced to a narrow flowering strip by mowing shortly before sampling. Irsch had the highest zOTU count and the lowest within‐site beta diversity. This suggests that the plant species were visited by many shared arthropod species, potentially because the flowering strip acted as a refuge from the mowing disturbance (Bruppacher et al., 2016). While similar refuge effects have been reported elsewhere (Buri et al., 2013; Valtonern et al., 2006; Wintergerst et al., 2021), our dataset does not allow us to conclusively confirm this mechanism, as we did not include comparable mown and unmown areas across multiple sites or sampling periods. Therefore, these management implications should be regarded as hypotheses requiring further empirical testing.

To find out whether arthropod communities are specialized on an even smaller scale than at the plant species level, different plant compartments were examined, showing that within a plant, the two compartments of flower and stem have clear differences in arthropod composition. This differentiation underscores the ecological specificity associated with different plant parts. For the flowers, arthropod communities varied distinctly between plant species, with flower color also having a minor influence on community composition. Since flowers differ more in structure, color, and size compared to green tissues (Benlloch et al., 2007), they likely provide a more specialized habitat and can attract specific arthropods through their odor and visual traits (Mulligan & Kevan, 1973; Randlkofer et al., 2010). Additionally, in grassland ecosystems, flowers tend to be spatially separated from one another, unlike green parts, which are often entangled with other plants. This separation may limit the movement of arthropods between flowers, leading to more plant‐specific communities on flowers. In contrast, the green tissues are more interconnected, potentially allowing easier transfer of arthropod species between plants. This suggests that plant specificity can play a stronger role in shaping communities associated with floral plant parts. This observation aligns with other studies that also focused primarily on flowers to demonstrate plant species‐driven arthropod clustering (Thomsen & Sigsgaard, 2019). Interestingly, even the stem exhibited plant‐specific arthropod compositions, indicating the pervasive influence of host species on community assembly. Knowing about such small‐scale differences in grassland ecosystems can help to better implement conservation measures and understand changes. Programs aiming to conserve arthropod diversity must also consider traits such as flowering time and habitat connectivity.

To deepen our understanding of the dynamics of arthropod communities, we examined patterns in herbivore and non‐herbivore communities, as their interactions with plants are fundamentally different. Although the difference in explained variance was moderate, herbivorous arthropods still showed clearer clustering by plant species than non‐herbivores, as evidenced by significant differences in beta diversity. This pattern likely reflects their dependency on specific host plants. It may also indicate coevolutionary dynamics whereby plants evolve defenses, such as secondary metabolites or structural barriers, and herbivores counter‐adapt to overcome these defenses, fostering specialization (Ehrlich & Raven, 1964; War et al., 2012). This evolutionary interplay often leads to a higher proportion of specialist herbivores tied to specific plants, as these species have adapted to metabolize unique defensive chemicals or exploit particular plant traits (Kant et al., 2015). This close relationship is also reflected in the pronounced decline in herbivore communities as a result of plant diversity loss (Fonseca, 2009; Uchida et al., 2016). By contrast, non‐herbivorous arthropods, which may use plants more opportunistically for shelter or hunting, exhibit less dependency on specific plant species. Still, not only are herbivorous arthropods found in grasslands, but also pollinator, predator, and decomposer species, which may be important for the health of grassland systems.

The ability to detect arthropod communities, especially with regard to arthropod–plant interactions, using eDNA metabarcoding simplifies monitoring and offers a robust complement to traditional methods (Weber et al., 2024). In particular, the localized nature of plant‐based eDNA can be of great benefit for the investigation of interaction relationships. We could detect not only plant‐specific arthropod assemblies but also community constellations at the different plant parts. Although we did not directly quantify airborne eDNA, the highly localized and distinct community patterns observed in this study suggest that the detected signals predominantly originate locally. Previous studies have shown that plant‐based arthropod eDNA is often highly specific to individual plant species and reflects direct biological interactions, including the detection of monophagous insect species on their known host plants (Stothut, Kühne, et al., 2024; Stothut, Mahla, et al., 2024; Thomsen & Sigsgaard, 2019; Weber et al., 2024). Consistent with this, we also detected monophagous taxa in association with their respective host plants, which underscores that much of the DNA likely originates from direct contact rather than aerial deposition. However, this conclusion should be drawn with caution, as airborne eDNA can still be deposited heterogeneously and may contribute to the overall signal to some extent (Lynggaard et al., 2022). Therefore, targeted controls and comparative studies are needed to distinguish local and non‐local traces of arthropod DNA detected on plants.

Furthermore, the detection of specialized herbivore–plant associations highlights the potential of eDNA to capture fine‐scale ecological interactions. Such intimate and specialized relationships emphasize the localized occurrence of eDNA and illustrate its utility for studying plant–arthropod networks. Together, these findings underline the evolutionary and ecological mechanisms shaping arthropod diversity and reinforce the value of eDNA metabarcoding as a tool for monitoring plant‐associated arthropod communities and informing biodiversity conservation efforts.

To further improve the method, especially in field applications, direct filtering using a portable filter system would simplify the workflow, particularly when handling large numbers of samples. This approach would reduce the number of plastic bags required for storage, as only the filter would need to be stored for subsequent laboratory processing. In addition, for simplifying the analysis of sequencing data, we recommend using pipelines such as APSCALE (Buchner et al., 2022), which have improved considerably since we completed our analyses. Such methodological optimizations may enable the application of this approach to a wider range of ecosystems, including forests or wetlands, and facilitate the investigation of important issues such as the effects of different land‐use strategies or the impact of invasive species.

## CONCLUSIONS

This study underscores the importance of plant species, site effects, and plant compartments as key drivers of arthropod community composition in grasslands. Arthropods exhibit notable specialization not only at the plant level but also within different plant parts, emphasizing the need to include detailed vegetation surveys in biodiversity monitoring programs. Herbivorous arthropods, in particular, are closely tied to specific plants, making them especially vulnerable to the loss of plant diversity. Our findings reinforce the potential of plant‐based eDNA metabarcoding as a powerful tool for monitoring and conserving arthropod biodiversity, providing critical insights for preserving grassland ecosystems in the face of insect decline.

## AUTHOR CONTRIBUTIONS

Lisa Mahla and Henrik Krehenwinkel conceived the ideas, conceptualized, and designed the survey. Lisa Mahla, Juliana Becker, Lea Groß, and Anna‐Sophie Tiltmann did the field work. Lisa Mahla, Juliana Becker, Lea Groß, Anna‐Sophie Tiltmann, and Susan Kennedy generated the data. Lisa Mahla analyzed the data. Lisa Mahla wrote the first draft. Lisa Mahla, Susan Kennedy, and Henrik Krehenwinkel reviewed and edited the manuscript.

## CONFLICT OF INTEREST STATEMENT

The authors declare no conflicts of interest.

## Supporting information


Appendix S1.

## Data Availability

Data (Mahla et al., 2025) are available in Figshare at https://doi.org/10.6084/m9.figshare.28062344.v1.
