# Supplementary material for: Plant‐derived environmental DNA reveals fine‐scaled community differentiation in grassland arthropods
Source: Ecol Appl. 2025 Dec 19;35(8):e70147. doi: 10.1002/eap.70147 (PMC12716967; doi:10.1002/eap.70147)
Supplement: Supplementary file 1 — Appendix S1. [file EAP-35-e70147-s001.pdf]

Appendix S1

Plant-derived environmental DNA reveals fine-scaled community differentiation in grassland arthropods

Lisa Mahla, Juliana Becker, Lea Groß, Anna-Sophie Tiltmann, Susan Kennedy, Henrik Krehenwinkel

Ecological Applications

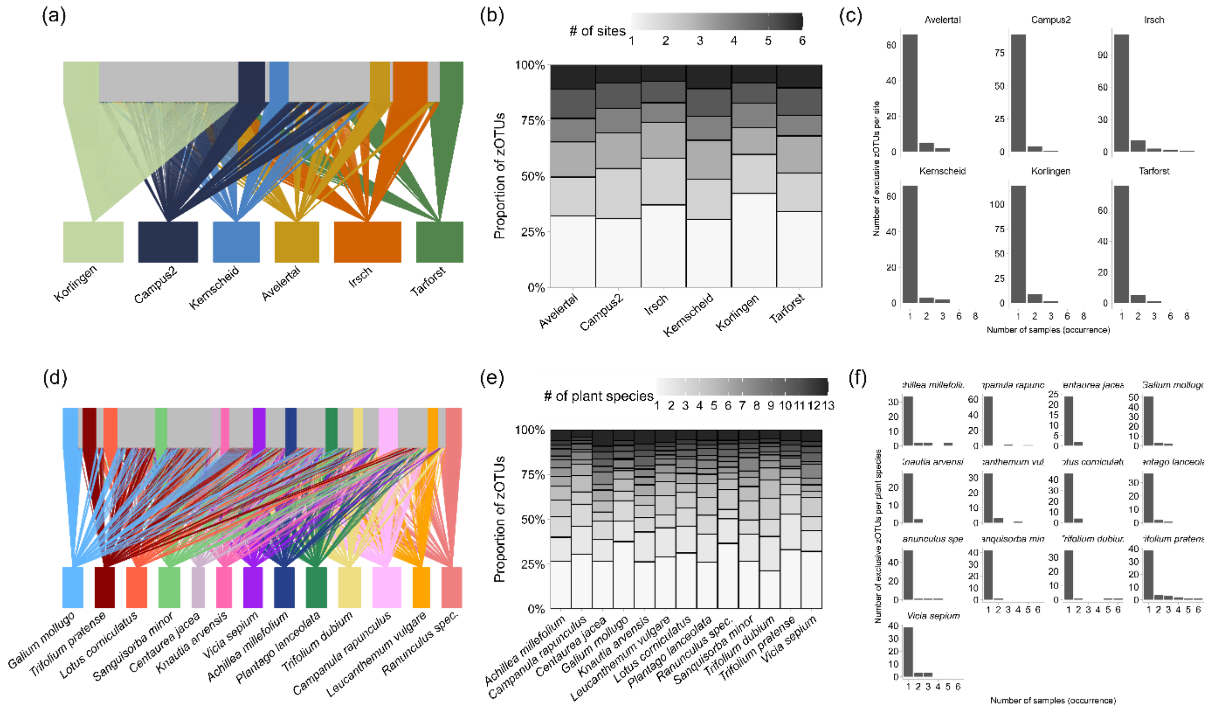

Figure S1:

Bipartite networks of sites (a) and the 13 plant species (d) show in the upper part the occurrence of different arthropod zOTUs and in the lower boxes the respective site or plant species. The coloured upper boxes show the number of taxa found only at the respective location or host plant coloured the same way. Grey boxes are taxa shared with different sites or plants. The 100% bar charts show the percentage of zOTUs for (b) each site or (e) each plant species being unique or shared among different numbers of sites/plant species. The gradient bar above each barplot shows in how many sites or species the zOTUs occur (“1” = zOTUs are unique to the site or species; “2” = zOTUs are shared between two sites/species; etc.). The bar charts show in how many samples those unique taxa occur for the sites (c) with 13 samples per site and the plant species (f) with 6 samples for each plant species.

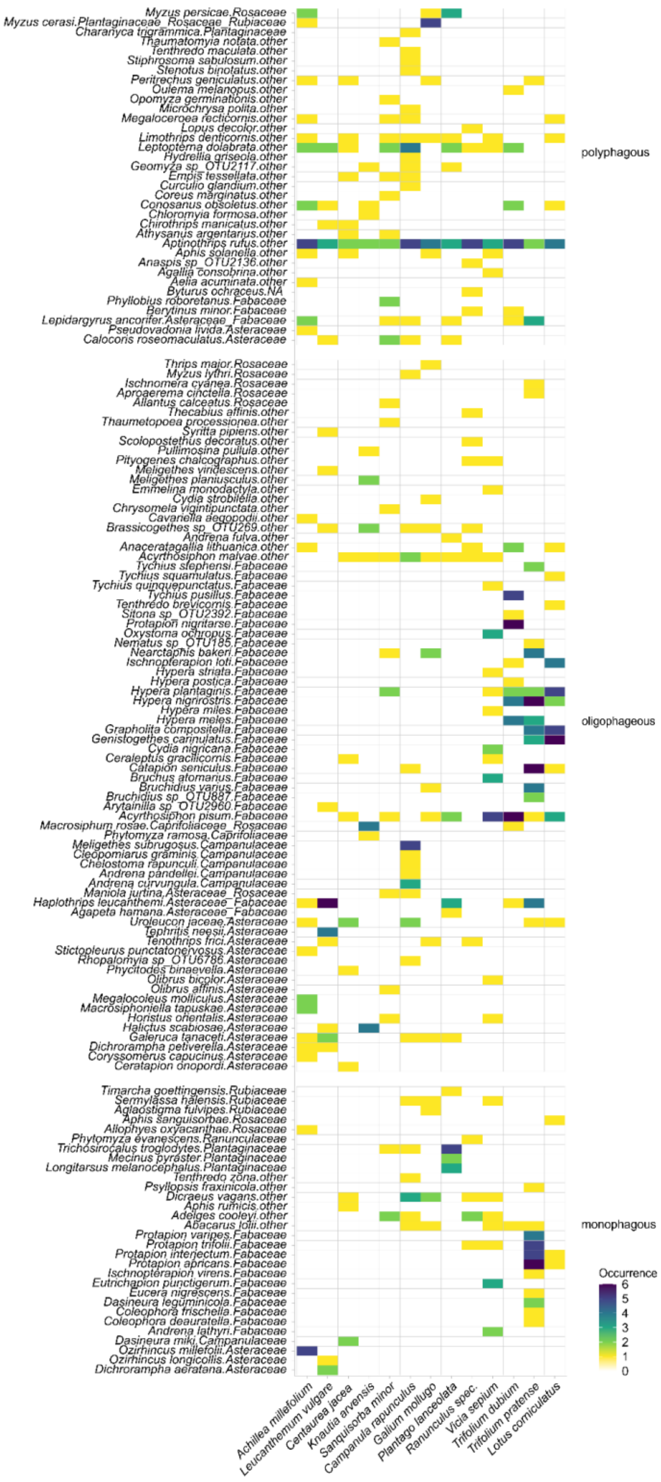

Figure S2:

Heat map of the occurrence of different arthropod species/taxa in each plant species with indication of the phagy type of this taxon (only for OTUs with 100 match to the reference database and where information on phagy could be determined). The darker the colour, the more frequently the taxon occurred in the samples of the plant species.

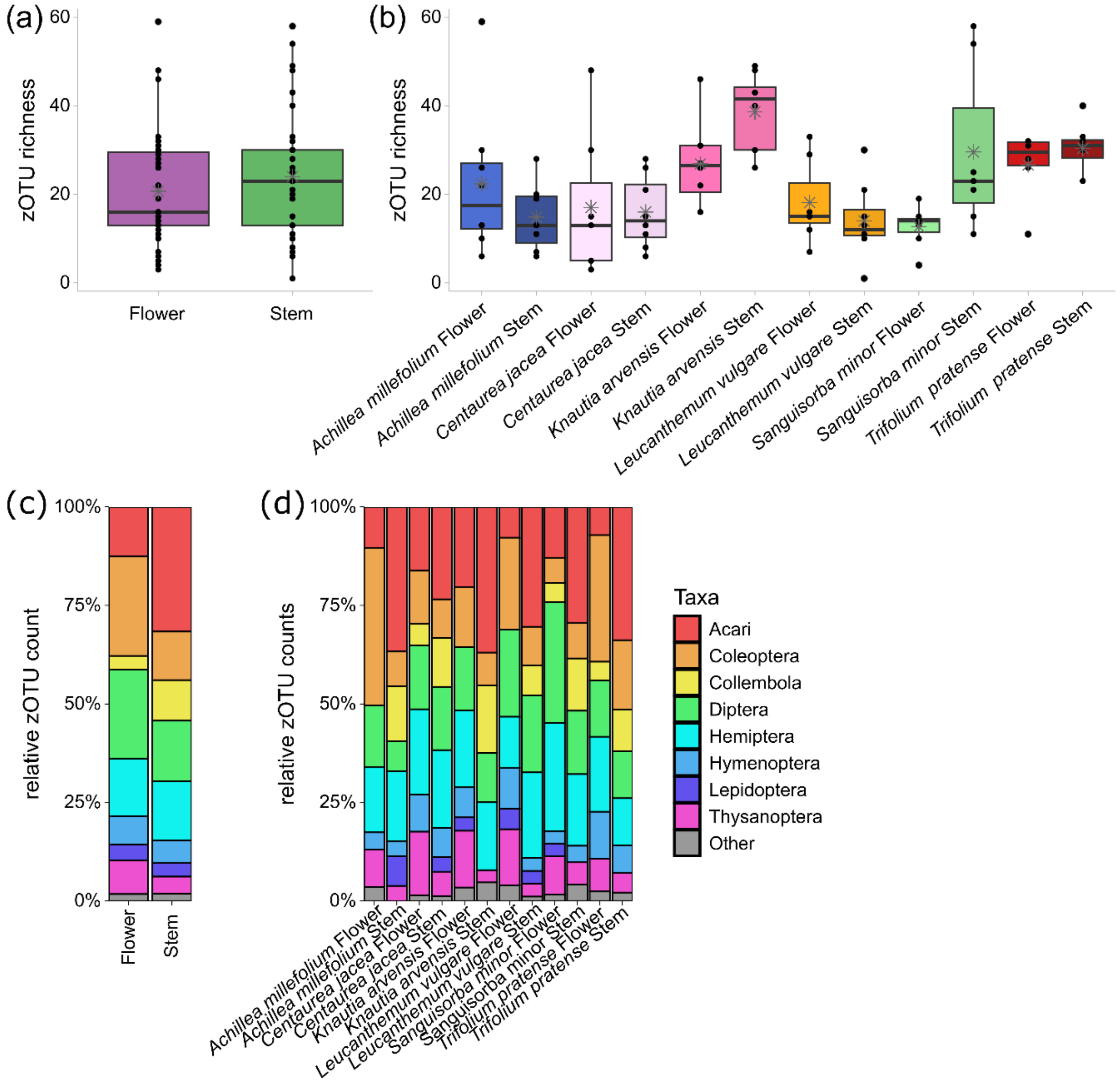

Figure S3:

zOTU richness and order/taxa composition of the flower and the stem samples. Boxplots with error bars and values for each sample as dots show the zOTU richness of (a) all flower and stem samples together and (b) all plant species separated. Order compositions are shown in 100% bar charts for (c) all flower and stem samples together and (d) each plant species separately. Colours represent orders/taxa with all orders below 3% combined in “Other”.

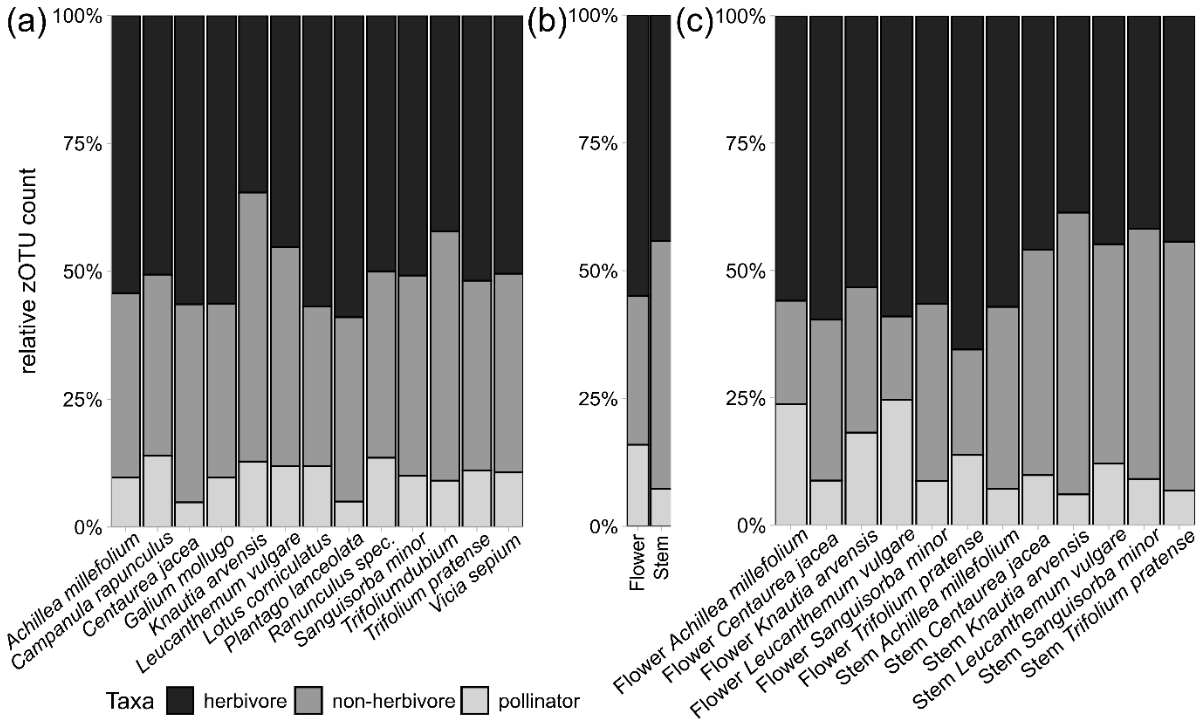

Figure S4:

100% bar chart for the determined herbivore and non-herbivore arthropods (zOTUs with >=98% match to the reference database) for (a) each of the complete collected plant species, (b) the flower and stem species combined, and (c) the flower and stem species separately. Non-herbivores in lighter grey are here split up into pollinators and other non-herbivores like predators and parasites. Herbivores contain mining, galling, plant chewing and sucking arthropods.

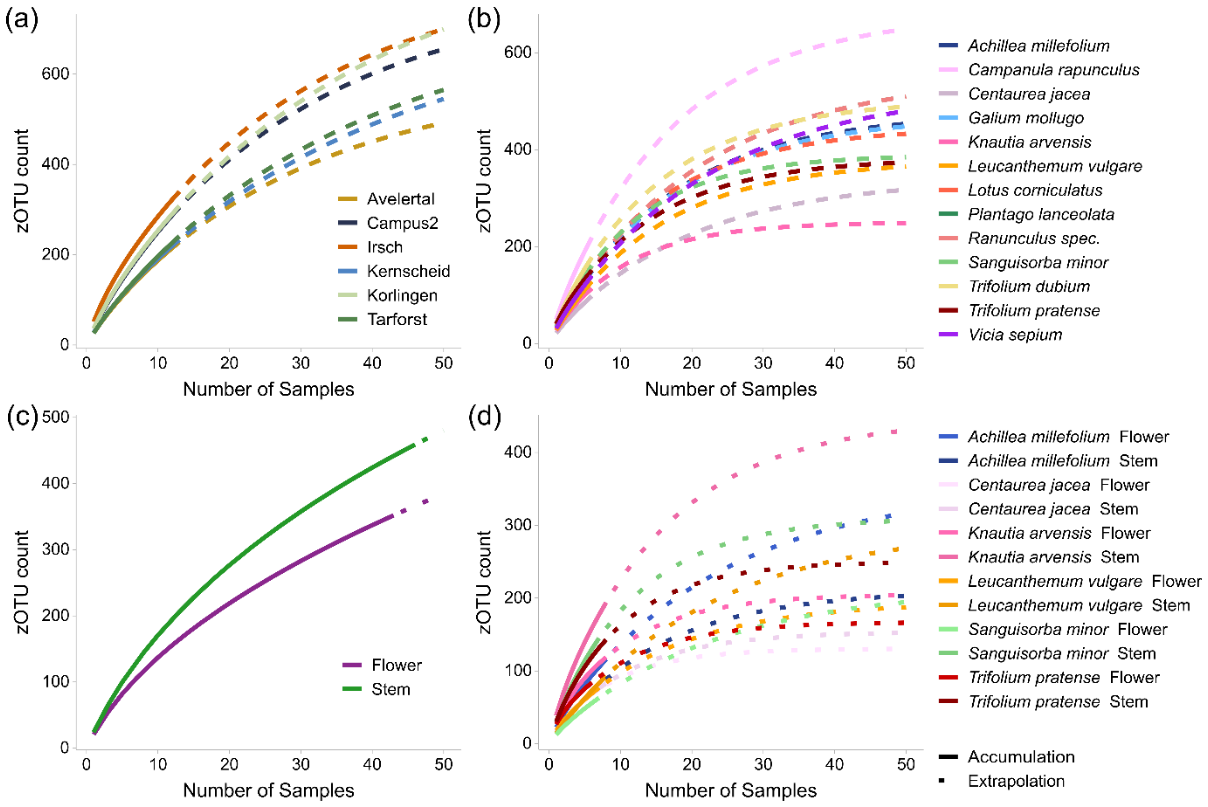

Figure S5:

Accumulation curves with the zOTU count per number of samples for the different sample types with extrapolation (dotted line) and the different (a) sites, (b) complete collected plant species, (c) the two compartments and (d) the different compartments per plant species separately.

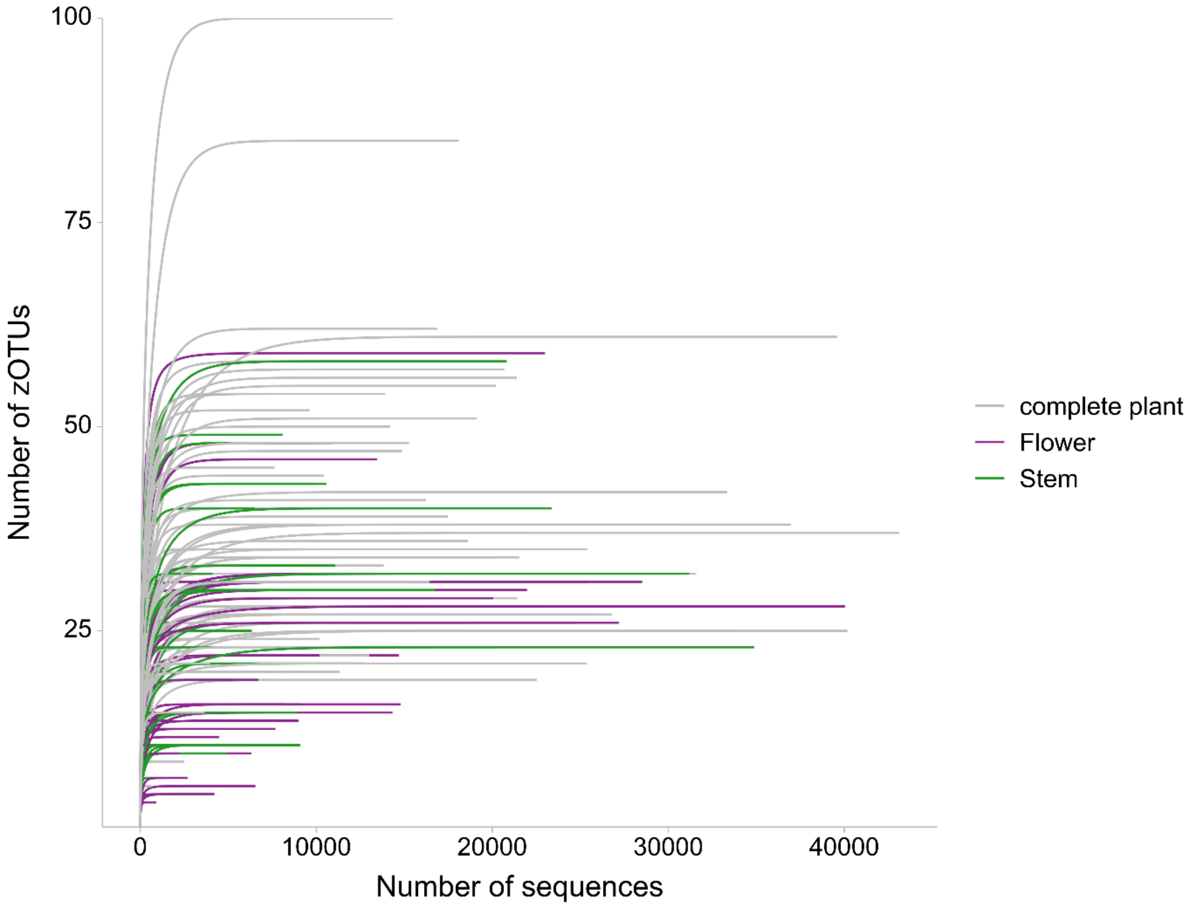

Figure S6:

Rarefection curves of the samples used in the study. Shows the number of zOTUs per number of sequences for whole plant specimens (complete plant), flower and stem samples.

Table S1:

Sampling site metadata. The complete area of the site, the real sampling range (which excludes inaccessible parts such as cordoned-off areas), altitude, landscape of close surroundings (buffer of 500m around the site) as well as the wider surroundings (buffer of 500m – 2 km around the site) and the different vegetation structures found at the sites compiled under openness of the site.

Site name	Avelertal	Campus2	Irsch	Kernscheid	Korrlingen	Tarforst
Max size (m^2)	30.733	53.771	15.722	118.206	109.072	55.816
Real sampling range (m^2)	30.700	53.700	15.700	80.000	109.000	48.300
Altitude above sea level (m)	215	258	257	264	377	277
Landscape in the close surrounding	Hedges	Road	Street	Field	Street	Hedge
Land use in the wider surrounding	Natural	Urban	Urban	Agrar	Agrar	Urban
Openness of the site	More than 10 bushes or trees	no higher structural vegetation	no higher structural vegetation	5-10 bushes or trees	5-10 bushes or trees	5-10 bushes or trees

Table S2:

Information about the plants sampled. Scientific name, plant family, flower colour, common growth height with specific categories (1= small to 4= very high) for this study, lifeform by Raunkiær and assignment to the two data sets/surveys that were compiled (whole plant specimens at all 6 sites and compartments at the two sites "Avelertal" and "Tarforst").

Plant name	Plant family	Flower colour	Growth hight in cm (height category)	Lifeform (Raunkiær)	Collected as whole plant specimens (at all 6 sites)	Collected as plant parts (at Avelertal, Tarforst)
Achillea millefolium L.	Asteraceae	white	High 20-60 (-80) (cat: 3)	Chamaephyt	Yes	Yes
Campanula rapunculus L.	Campanulaceae	violette	Very high, 50-80 (cat: 4)	Hemicryptophyte	Yes	No
Centaurea jacea L.	Asteraceae	violette	High, 15 – 60 (cat: 3)	Hemicryptophyte	Yes	Yes
Galium mollugo agg.	Rubiaceae	white/ yellow	High, 30-100 (cat: 3)	Hemicryptophyte	Yes	No
Knautia arvensis (L.) Coult., s. str	Caprifoliaceae	violette	Very high, 30 -80 (cat: 4)	Hemicryptophyte	Yes	Yes
Leucanthemum vulgare agg.	Asteraceae	white	Very high, 20-80 (-100) (cat: 4)	Hemicryptophyte	Yes	Yes
Lotus corniculatus L.	Fabaceae	yellow	Small, 20-30 (cat: 1)	Hemicryptophyte	Yes	No
Plantago lanceolata L.	Plantaginaceae	white-brown	Medium, 10cm-50 (cat: 2)	Hemicryptophyte	Yes	No
Ranunculus spec.	Ranunculaceae	yellow	Medium, 15-35 (cat: 2)	Geophyt	Yes	No
Sanguisorba minor Scop.	Rosaceae	brown-red	High 15-50(-80) (cat: 3)	Hemicryptophyte	Yes	Yes
Trifolium dubium Sibth.	Fabaceae	yellow	Small, 10cm - 25 (cat: 1)	Therophyt	Yes	No
Trifolium pratense L.	Fabaceae	violette	Medium, 5cm -80 (cat: 2)	Hemicryptophyte	Yes	Yes
Vicia sepium L.	Fabaceae	violette	Medium, 30-60 (cat: 2)	Hemicryptophyte	Yes	No

Table S3:

R^2 values of the adonis2 calculation for the herbivore and non-herbivore arthropod communities for different plant attributes, sites and plant species. Information about the different attributes are given in Appendix S1: Table S2. Only R^2 values of significant adonis2 calculation are included ($p<0.05$) with method = "jaccard", and p.adjust.methods = "holm".

	Herbivore			Non-herbivore		
	Whole plant specimens	flower	stem	Whole plant specimens	flowers	stem
Plant species	0.25161	0.24746	0.16533	0.20394	0.18169	0.14750
Site	0.07427	0.03008	0.03190	0.07274		0.04099
Colour	0.09461	0.13702	0.06534	0.08625	0.09662	0.05974
Lifeform	0.07647	0.08133	0.02997	0.06854		
height category	0.02793	0.05094	0.05660		0.05402	0.04945

Table S4:

Results of the pairwise adonis (pairwise.adonis) calculation for the different compartments for both the herbivore and non-herbivore arthropod communities between each plant species. Herbivores include mining, galling, plant chewing and sucking arthropods while non-herbivores include pollinators, predators and other arthropod types. The calculation was done with sim.method= "jaccard" and the default settings. Df= Degrees of freedom, sums of sqs = Sums of squares, F-Model, R2, adjusted P-value (p.adjusted) and significance sign (. = p<0.1) are given for each pair.

Pairwise adonis calculation for herbivore arthropod communities of stem samples						
pairs	Df	Sums of sqs	F-Model	R2	p.adjusted	sig
Achillea millefolium vs *Centaurea jacea*	1	0,664592	1,416585	0,105585	0,015	.
Achillea millefolium vs *Knautia arvensis*	1	0,641088	1,396207	0,096984	0,195	
Achillea millefolium vs *Leucanthemum vulgare*	1	0,565452	1,166982	0,095914	0,195	
Achillea millefolium vs *Sanguisorba minor*	1	0,483415	1,036931	0,079538	1	
Achillea millefolium vs *Trifolium pratense*	1	0,728986	1,607007	0,110016	0,045	.
Centaurea jacea vs *Knautia arvensis*	1	0,715778	1,599103	0,109534	0,435	
Centaurea jacea vs *Leucanthemum vulgare*	1	0,633236	1,344762	0,108934	0,3	
Centaurea jacea vs *Sanguisorba minor*	1	0,699688	1,542239	0,113884	0,21	
Centaurea jacea vs *Trifolium pratense*	1	0,858365	1,941659	0,129949	0,015	.
Knautia arvensis vs *Leucanthemum vulgare*	1	0,679413	1,477222	0,109609	0,105	
Knautia arvensis vs *Sanguisorba minor*	1	0,540153	1,214137	0,085418	1	
Knautia arvensis vs *Trifolium pratense*	1	0,925897	2,129781	0,13204	0,015	.
Leucanthemum vulgare vs *Sanguisorba minor*	1	0,6537	1,397782	0,112745	0,09	
Leucanthemum vulgare vs *Trifolium pratense*	1	0,720845	1,588007	0,116868	0,03	.
Sanguisorba minor vs *Trifolium pratense*	1	0,816818	1,859142	0,125118	0,045	.
Pairwise adonis calculation for non-herbivore arthropod communities of stem samples						
pairs	Df	Sums of sqs	F-Model	R2	p.adjusted	sig

Achillea millefolium vs *Centaurea jacea*	1	0,403025	0,852709	0,061555	1	
Achillea millefolium vs *Knautia arvensis*	1	0,603025	1,34609	0,09383	0,87	
Achillea millefolium vs *Leucanthemum vulgare*	1	0,476524	0,995322	0,071118	1	
Achillea millefolium vs *Sanguisorba minor*	1	0,641146	1,381522	0,103241	0,09	
Achillea millefolium vs *Trifolium pratense*	1	0,731782	1,629283	0,111371	0,015	.
Centaurea jacea vs *Knautia arvensis*	1	0,653041	1,444615	0,093535	0,18	
Centaurea jacea vs *Leucanthemum vulgare*	1	0,517576	1,07686	0,071425	1	
Centaurea jacea vs *Sanguisorba minor*	1	0,576867	1,234653	0,086736	1	
Centaurea jacea vs *Trifolium pratense*	1	0,752166	1,659934	0,105999	0,015	.
Knautia arvensis vs *Leucanthemum vulgare*	1	0,637212	1,392092	0,090442	0,405	
Knautia arvensis vs *Sanguisorba minor*	1	0,68124	1,539277	0,10587	0,45	
Knautia arvensis vs *Trifolium pratense*	1	0,845319	1,964793	0,12307	0,015	.
Leucanthemum vulgare vs *Sanguisorba minor*	1	0,652753	1,378999	0,095904	0,015	.
Leucanthemum vulgare vs *Trifolium pratense*	1	0,758133	1,652372	0,105567	0,015	.
Sanguisorba minor vs *Trifolium pratense*	1	0,556847	1,254916	0,088034	1	
Pairwise adonis calculation for herbivore arthropod communities of flower samples						
pairs	Df	Sums of sqs	F-Model	R2	p.adjusted	sig
Achillea millefolium vs *Centaurea jacea*	1	1,287832	3,683905	0,234884	0,03	.
Achillea millefolium vs *Knautia arvensis*	1	1,264634	3,361362	0,193612	0,015	.
Achillea millefolium vs *Leucanthemum vulgare*	1	1,262782	3,623839	0,21799	0,015	.
Achillea millefolium vs *Sanguisorba minor*	1	1,264293	3,588051	0,216303	0,015	.
Achillea millefolium vs *Trifolium pratense*	1	1,51503	4,852034	0,28792	0,015	.
Centaurea jacea vs *Knautia arvensis*	1	0,58695	1,273742	0,09596	0,855	
Centaurea jacea vs *Leucanthemum vulgare*	1	0,827639	1,899614	0,147261	0,03	.
Centaurea jacea vs *Sanguisorba minor*	1	0,728512	1,654607	0,130751	0,24	
Centaurea jacea vs *Trifolium pratense*	1	0,900374	2,245615	0,183381	0,135	
Knautia arvensis vs *Leucanthemum vulgare*	1	0,770684	1,708327	0,116147	0,015	.

Knautia arvensis vs *Sanguisorba minor*	1	0,539459	1,185544	0,083574	1	
Knautia arvensis vs *Trifolium pratense*	1	0,882843	2,084781	0,148017	0,03	.
Leucanthemum vulgare vs *Sanguisorba minor*	1	0,849193	1,967899	0,140887	0,03	.
Leucanthemum vulgare vs *Trifolium pratense*	1	1,069287	2,707353	0,197511	0,03	.
Sanguisorba minor vs *Trifolium pratense*	1	1,010394	2,528754	0,186917	0,03	.
Pairwise adonis calculation for non-herbivore arthropod communities of flower samples						
pairs	Df	Sums of sqs	F-Model	R2	p.adjusted	sig
Achillea millefolium vs *Centaurea jacea*	1	0,747995	1,778892	0,139205	0,06	
Achillea millefolium vs *Knautia arvensis*	1	0,652358	1,439197	0,099673	0,045	.
Achillea millefolium vs *Leucanthemum vulgare*	1	0,505481	1,087588	0,083101	1	
Achillea millefolium vs *Sanguisorba minor*	1	0,623228	1,352563	0,101296	1	
Achillea millefolium vs *Trifolium pratense*	1	0,883475	2,212468	0,167453	0,195	
Centaurea jacea vs *Knautia arvensis*	1	0,720941	1,843895	0,133192	0,465	
Centaurea jacea vs *Leucanthemum vulgare*	1	0,846522	2,127668	0,162075	0,105	
Centaurea jacea vs *Sanguisorba minor*	1	0,319752	0,812577	0,068789	1	
Centaurea jacea vs *Trifolium pratense*	1	0,322691	1,011026	0,091819	1	
Knautia arvensis vs *Leucanthemum vulgare*	1	0,586345	1,350593	0,094114	1	
Knautia arvensis vs *Sanguisorba minor*	1	0,589115	1,368604	0,09525	1	
Knautia arvensis vs *Trifolium pratense*	1	0,824493	2,218856	0,15605	0,12	
Leucanthemum vulgare vs *Sanguisorba minor*	1	0,671928	1,526969	0,112883	0,93	
Leucanthemum vulgare vs *Trifolium pratense*	1	1,026566	2,725182	0,198553	0,045	.
Sanguisorba minor vs *Trifolium pratense*	1	0,423492	1,137393	0,09371	1	
